# Fabrication of Conductive Tissue Engineering Nanocomposite Films Based on Chitosan and Surfactant-Stabilized Graphene Dispersions

**DOI:** 10.3390/polym14183792

**Published:** 2022-09-10

**Authors:** Aleksandr S. Buinov, Elvira R. Gafarova, Ekaterina A. Grebenik, Kseniia N. Bardakova, Bato Ch. Kholkhoev, Nadezhda N. Veryasova, Pavel V. Nikitin, Nastasia V. Kosheleva, Boris S. Shavkuta, Anastasia S. Kuryanova, Vitalii F. Burdukovskii, Peter S. Timashev

**Affiliations:** 1 Baikal Institute of Nature Management, Siberian Branch, Russian Academy of Sciences, Marii Sakh’yanovoi St. 6, 670047 Ulan-Ude, Russia; 2 Institute for Regenerative Medicine, Sechenov University, Trubetskaya St. 8-2, 119991 Moscow, Russia; 3 World-Class Research Center “Digital Biodesign and Personalized Healthcare”, Sechenov First Moscow State Medical University, Trubetskaya St. 8-2, 119991 Moscow, Russia; 4 Institute of Photonic Technologies, Federal Scientific Research Centre “Crystallography and Photonics”, Russian Academy of Sciences, Pionerskaya St. 2, 142190 Troitsk, Russia; 5 FSBSI “Institute of General Pathology and Pathophysiology”, Baltiyskaya St. 8, 125315 Moscow, Russia; 6 Semenov Federal Research Center of Chemical Physics, Russian Academy of Sciences, Kosygina St. 4, 119991 Moscow, Russia; 7 Chemistry Department, Lomonosov Moscow State University, Leninskiye Gory 1-3, 119991 Moscow, Russia

**Keywords:** chitosan, graphene, biomaterials, electrical conductivity, cellular adhesion, inflammatory reaction

## Abstract

Chitosan (CS)/graphene nanocomposite films with tunable biomechanics, electroconductivity and biocompatibility using polyvinylpyrrolidone (PVP) and Pluronic F108 (Plu) as emulsion stabilizers for the purpose of conductive tissue engineering were successfully obtained. In order to obtain a composite solution, aqueous dispersions of multilayered graphene stabilized with Plu/PVP were supplied with CS at a ratio of CS to stabilizers of 2:1, respectively. Electroconductive films were obtained by the solution casting method. The electrical conductivity, mechanical properties and in vitro and in vivo biocompatibility of the resulting films were assessed in relation to the graphene concentration and stabilizer type and they were close to that of smooth muscle tissue. According to the results of the in vitro cytotoxicity analysis, the films did not release soluble cytotoxic components into the cell culture medium. The high adhesion of murine fibroblasts to the films indicated the absence of contact cytotoxicity. In subcutaneous implantation in Wistar rats, we found that stabilizers reduced the brittleness of the chitosan films and the inflammatory response.

## 1. Introduction

Despite the progress in the field of high-tech medical care, myocardial infarction still remains the main etiological factor for the development of heart failure. Due to the minimal regenerative potential of cardiomyocytes, the heart is almost unable to repair itself [1]. The death of cardiomyocytes is accompanied by the focal block of conduction and contractility, which ultimately leads to a decrease in cardiac output [2]. One of the most promising approaches for correcting heart function and improving the quality of a patients’ life is cardiac tissue engineering. A large number of scaffolds are currently being developed for the purpose of the reconstructive surgery of a wide range of tissues and organs. Scaffolds serve as an adhesive substrate and as physical support to form new tissues through the navigation of cell growth and differentiation. A variety of decellularized tissues [3,4,5], biocompatible natural (collagen, cellulose, fibronectin, fibroin, etc.) [6,7,8,9] and synthetic polymers (polylactide, polyglycolide, polycaprolactone, polyvinyl alcohol, etc.) [10,11,12,13] are used to manufacture scaffolds. The choice of a suitable material is one of the fundamental factors of success. An ideal cardiac patch must reproduce key structural and functional properties of native heart tissue including mechanical strength and electroconductivity. These microenvironmental signals influence the proliferation of precursor cells and their differentiation to cardiomyocytes.

Chitosan (CS) is widely applied in tissue engineering. According to the literature, scaffolds based on CS enhance the cardiogenic phenotype and expression of cardiac markers following cultivation without external electrical stimulation [14]. However, its main disadvantage is low mechanical strength [15], which is solved by obtaining composite materials [16,17], chitosan derivatives [18,19,20,21], or using biocompatible fillers [22,23,24,25,26,27,28]. The latter approach is the most straightforward and creates wide opportunities to obtain scaffolds with required characteristics.

**Figure 1:**
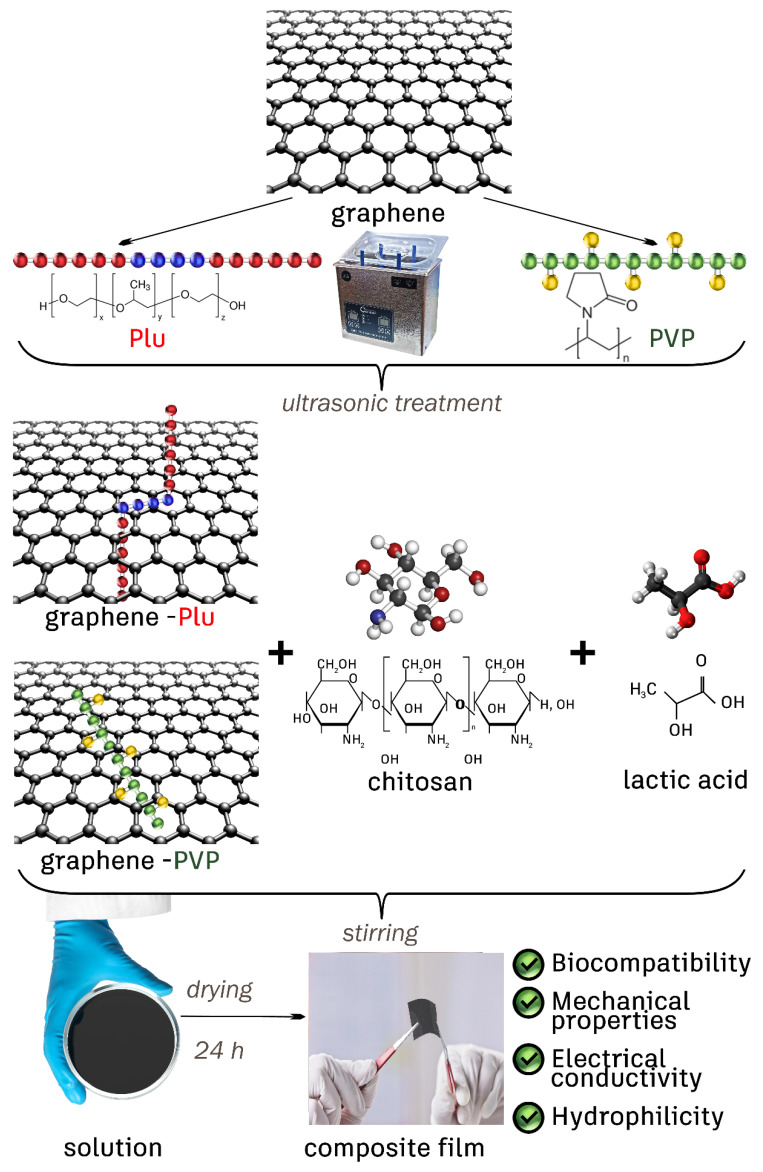
Preparation of graphene dispersions and fabrication of composite films.

Graphene is one of the most promising biocompatible fillers owing to its outstanding properties including high mechanical strength, electrical conductivity, large specific surface area, etc., [29,30]. The use of graphene as a filler makes it possible to render the host biomaterial with electrical conductivity, improved mechanical strength, cellular adhesion, proliferation and differentiation [31,32]. In the case of the regeneration of nervous and smooth muscle tissues of the heart, the presence of electrical conductivity is the key property that enables the electrostimulation of neurons and cardiomyocytes [33,34]. However, a direct assembly of a hydrophobic graphene with a hydrophilic polymer matrix of chitosan is complicated. Therefore, the hydrophilic derivative of graphene, graphene oxide (GO), devoid of electrical conductivity [35], is commonly utilized. The transformation of GO into electrically conductive reduced graphene oxide (rGO) is complicated by the cytotoxicity of the most reducing agents suitable for this purpose (sodium borohydride, hydrazine, 1,1-dimethylhydrazine, hydroquinone) [36,37,38]. Therefore, it is necessary to develop new methods for introducing graphene into biopolymers to obtain electrically conductive scaffolds. This work demonstrates the preparation of electrically conductive biomaterials based on CS and graphene using stabilizing surfactants: polyvinylpyrrolidone (PVP) and Pluronic F108 (Plu). The general scheme of obtaining graphene dispersions and chitosan composites based on them is shown in Figure 1. The influence of the type of stabilizer on the formation of graphene dispersions and on the mechanical properties of composite materials was thoroughly discussed. The change of electrical conductivity depending on the graphene content in composites was also evaluated. A comprehensive biocompatibility study was performed including in vitro cytotoxicity testing and subcutaneous implantation in rats. The study highlights the potential of electroactive graphene-based constructions as a candidate material for cardiac tissue engineering. Such materials can be used to create 2D or 3D scaffolds for replacing of heterogeneity areas formed by fibrous tissue in postinfarction cardiosclerosis.

## 2. Materials and Methods

### 2.1. Reagents

In this work, CS (M = 200,000, degree of deacetylation 91%, Bioprogress, Moscow, Russia), PVP (M = 40,000, Sigma-Aldrich, St. Louis, MO, USA), Plu (M = 14,600, Sigma-Aldrich, St. Louis, MO, USA) and lactic acid (85%, Reagent, Moscow, Russia) were used as received. Multilayer graphene (MLG, 10–15 layers, interplanar spacing up to 3.49 A) was synthesized in the mode of thermal shock of intercalated compounds of fluorinated graphite C_2_F⋅xClF_3_ [39].

### 2.2. Composite Film Fabrication

Electrically conductive biomaterials were obtained in the form of films by the solution casting method. Graphene dispersions were obtained by dispersing MLG in an aqueous solution of stabilizers using a Sapphire (Soltec, Moscow, Russia) ultrasonic bath at a power of 100 W and a frequency of 35 kHz, with duration of ultrasonic treatment of Plu-stabilized dispersions being 1–2 h and in case of those PVP-stabilized, being 2–4 h. Next, 20 mL of the graphene dispersions with 0.3 mL of lactic acid were supplied with 370–400 mg of CS followed by 1–4 h dissolving (Supplementary Table S1). Then the composite solution was poured onto a polystyrene Petri dish and dried at room temperature for 24 h. Thus, we prepared the films containing graphene concentrations falling in the range from 1 to 4 wt.% after PVP-assisted preparation and from 1 to 5 wt.% after Plu-assisted preparation.

### 2.3. Transmission Electron Microscopy (TEM)

TEM images were obtained by a JEM-2200FS transmission electron microscope (JEOL Ltd., Tokyo, Japan) with a resolution of 0.1 nm at an accelerating voltage of 200 kV. Aqueous dispersions of graphene were deposited onto a copper grid covered with a carbon film.

### 2.4. Scanning Electron Microscopy (SEM)

SEM images were acquired by a tabletop scanning electron microscope TM3000 (Hitachi, Tokyo, Japan) at an accelerating voltage of 5 kV. The films of 0.4 × 0.4 cm^2^ were previously conditioned in desiccators containing phosphorus pentoxide P_2_O_5_ (25 °C) for 3 weeks. Pieces of film were fractured in liquid nitrogen.

### 2.5. Uniaxial Tensile Testing

The macromechanical characteristics of the films were determined using an Instron 3367 testing machine (Instron, Norwood, MA, USA). The films with the dimensions of 40 × 5 × 0.12 mm^3^ were soaked with phosphate-saline buffer solution (PBS, EcoService, Saint Petersburg, Russia). Both ends of the samples were fixed tightly in the clamps of the tensiometer followed by gradual stretching at a stretching rate of 10 mm/min and 1 N load cell at room temperature and a humidity of ~50% and recording the data on a personal computer.

### 2.6. Nanoindentation

Local mechanical characteristics (Young’s modulus) of the films with the size 0.5 × 0.5 cm^2^ were established using a Piuma Nanoindenter (Optics 11, Amsterdam, The Netherlands) in PBS. A probe with a cantilever stiffness of 26.5 N/m and radius of curvature of the spherical tip of 26 μm was used for indentation. During the measurements, the probe was always located inside the fluid medium at a sufficient depth, in order to avoid measurement errors due to adhesion forces at the air–water boundary. The samples were immobilized at the bottom of Petri dish to prevent floating. To measure the Young’s modulus the probe was dipped for 5 μm into the sample at each point of measurement. The Young’s modulus for each point was computed according to the Hertzian contact mechanics model for a spherical body indenting a flat surface, using the built-in Piuma software. The indentation area was 2500 μm × 2500 μm with a step size along the axes X and Y of 250 µm.

### 2.7. Electrical Conductivity

The electrical conductivity of the films was measured by the standard four-electrode method using an AVM-455 multimeter (Actacom, Moscow, Russia). Electrical contacts were made of silver paste. Lead wires were copper. The resulting films were measured by a micrometer and the thickness of the films varied from 100–150 µm. The films were cut to squares about 1 cm^2^ in order to make experimental samples.

### 2.8. Phase Analysis by X-ray Diffraction (XRD)

XRD was performed using a Bruker D8 Advance (Bruker AXS, Karlsruhe, Germany) diffractometer (CuK α-radiation) at a wavelength of 0.15406 nm.

### 2.9. Static Contact Angle Measurement

The static contact angle measurement of liquid droplets was performed using the Acam-MSC01 (Apex Instruments, West Bengal, India). The films of 1.0 × 1.0 cm^2^ soaked in water were placed on a glass slide and dried in a thermostat at 37 °C for 3 h. Distilled water and ethylene glycol were used as test liquids. Measurements were performed at different sites of the samples at 37 °C in three replications. The calculation of free surface energy was carried out by the Owens–Wendt–Rabel–Kjelble (OWRK) method according to the Equation:

(1) $$\frac{\sigma_{L}(1+\cos\theta )}{2\sqrt{\sigma_{L}^{D}}}=\sqrt{\sigma_{S}^{D}}+\sqrt{\sigma_{S}^{P}}\sqrt{\frac{\sigma_{L}^{P}}{\sigma_{L}^{D}}}$$

where *σ^P^* is polar and *σ^D^* is dispersive component of a surface energy, *θ* is the wetting angle of the solid surface with the test liquid and subscripts *S* and *L* indicate that this characteristic refers to a solid or liquid, respectively. 

### 2.10. MTT Test

MTT test was performed in order to assess the effect of soluble film components on cell viability. The film fragments with a total area of 6 cm^2^ from each sample group were incubated for 24 h in 1 mL of cell culture medium (DMEM/F12 (Gibco, Grand Island, NY, USA) with addition of 100 U/mL streptomycin, 100 g/mL penicillin, 1% GlutaMAX (Gibco, USA) and 5% fetal bovine serum (HyClone, Logan, UT, USA)) under an atmosphere containing 5% CO_2_ at 37 °C. Serial dilutions of the extracts in the medium were added to triplicate wells to the subconfluent monolayer of 3T3 murine fibroblasts cultured in a 96-well plate. Sodium dodecyl sulfate (SDS, Sigma-Aldrich, St. Louis, MO, USA) was added to separate wells as a positive (toxic) control and culture medium alone was a negative control. The plates were incubated for additional 24 h in a CO_2_-incubator at 37 °C. The medium was then replaced with 100 μL MTT solution (0.5 mg/mL in the additive-free DMEM/F12 medium) and incubated in a CO_2_ incubator at 37 °C for 3 h. After discarding the MTT solution, 100 μL aliquots of dimethyl sulfoxide were added to all wells and stirred. Cell viability was quantified by measuring the optical density of the solution at wavelengths of 567 nm and 630 nm on a Multiscan FC spectrophotometer (ThermoScientific, Waltham, MA, USA).

### 2.11. Contact Cytotoxicity

Confluence and cell viability on the surface-seeded films with a total area of 6 cm^2^ were investigated by differential staining of living and dead cells with a Live/Dead staining kit (Invitrogen, Waltham, MA, USA). Murine fibroblasts of the 3T3 line were stained after 76 h of incubation on the surface of the films. A scanning laser confocal microscope LSM 880 Airyscan (Carl Zeiss, Oberkochen, Germany) equipped with an AiryScan module and a GaAsP detector (Carl Zeiss, Oberkochen, Germany) was used to visualize green living and red dead cells. Z scans were obtained using an EC Plan-Neofluar lens (Carl Zeiss, Oberkochen, Germany) and lasers with wavelengths of 488 and 561 nm.

### 2.12. Subcutaneous Implantation in Rats

Tissue reaction was assessed by subcutaneous implantation in male Wistar line rats (age = 6 weeks, average weight = 180–200 g). The animals were divided into five groups (*n* = 5). The samples were disinfected with 70% ethanol for 30 min. All in vivo procedures and experiments were carried out in accordance with the recommendations for animal care and approved by the local ethical committee of Sechenov University (#82 from 07.02.2022, Moscow, Russia). Indoor acclimatization and preparation of animals for operational interventions was carried out for 14 days prior to the surgery. After that, the rats were anesthetized by intramuscular injection of zoletil (15 mg/kg, Vibrac, France) and xylazine (1 mg/kg, Interchemie, Venray, The Netherlands). In a preliminarily trimmed interscapular region, a 2-cm-long skin incision was performed with a scalpel in aseptic conditions. The subcutaneous fat was dissected in a blunt manner in the directions towards the right and left scapula, with the creation of two 2-cm-deep pockets. The resulting cavity was washed with an aqueous chlorhexidine solution. A sterile film fragment of 1.0 × 0.5 cm^2^ was sutured to the tissues with prolen 4.0. In order to prevent purulent-septic complications after surgery, antibiotic therapy with 2.5% enrofloxacin (5 mg/kg, Bayer, Leverkusen, Germany) was carried out for 7 days. The overall condition of the animals, the body temperature and the condition of the skin above the implantation area were evaluated in dynamics. Four weeks after the implantation, the animals were devitalized by inhalation of carbon dioxide. Two 0.5-cm-thick tissue fragments composed of implants separated along with the capsules formed around them, muscle tissues and adjacent areas of skin were explanted from each rat. The explants were washed in physiological saline and fixed in 10% buffered formalin.

### 2.13. Histological Analysis

Tissue samples were dehydrated and poured into paraffin blocks using a standard procedure. All samples underwent cross-sectional microtome sectioning with the step size of 4–5 μm (8 slices per block) using the Sakura Accu-Cut SRM 200 microtome (Sakura Finetek, Torrance, CA, USA). The deparaffinized sections were dehydrated in a battery of increasing concentrations of alcohols and stained with hematoxylin and eosin. After embedding into the synthetic medium of Shandonmount TM (USA), the samples were studied by bright-field, phase-contrast, dark-field and polarized light microscopy. Examination, analysis and photography of histological preparations were carried out using a LEICA DM4000 B LED microscope equipped with 45 LEICA DFC7000 T digital video cameras and LAS V4.8 software (Leica Microsystems, Heerbrugg, Switzerland).

Immunofluorescence research was used to profile leukocyte infiltrates. Serial sections with a thickness of 3 micrometers were made from the paraffin-embedded tissue samples. The samples were then deparaffinized and rehydrated by sequentially immersing the slides in xylene, 100%, 95%, 70% and 50% ethanol and deionized water. The sections were dried in a thermostat at 45 °C. In order to demask antigens, we transferred the slides in boiling sodium citrate buffer, pH 6.0, securing the lid on top. In 3 min, the heater was turned off and placed in an empty sink, after which the pressure relief valve was activated and cold water was poured into the heater for 10 min. Sections were dried and then incubated with rabbit monoclonal antibodies against CD4+ T lymphocytes (Sigma-Aldrich, St. Louis, MO, USA) conjugated with Alexa Fluor 594, rabbit monoclonal antibodies against CD8+ T lymphocytes (Sigma-Aldrich, St. Louis, MO, USA) conjugated with Alexa Fluor 488, rabbit anti-CD68 monoclonal antibodies against macrophages (Sigma-Aldrich, St. Louis, MO, USA) conjugated with Alexa Fluor 633, rabbit anti-CD20 monoclonal antibodies against B lymphocytes (Sigma-Aldrich, St. Louis, MO, USA) conjugated with Alexa Fluor 568 and rabbit anti-CD138 monoclonal antibodies against plasma cells (Sigma-Aldrich, St. Louis, MO, USA) conjugated with Alexa Fluor 405 diluted in 1% bovine serum albumin in PBS at room temperature for 1–2 h. After that, the samples were kept overnight at a temperature of 4 °C in a humid chamber. The sections were then washed twice with 1% bovine serum albumin in PBS/Tween-20 solution for 10 min. The DAPI dye was then applied. After incubation with the dye, the slides were washed for 10 min. The preparations were digitized using the Aperio VERSA glass scanner (Leica, Wetzlar, Germany) in fluorescent mode. In order to compare the average values of the number of different types of cells, the Mann–Whitney U-test was used. Differences were considered significant at *p* < 0.05.

### 2.14. Polymerase Chain Reaction

Total RNA was isolated from the obtained tissue using the RNeasy Mini Kit (Qiagen, Hilden, Germany). The analysis of the expression of the CD4, CD8 and CD19 genes was performed using the method of determining the threshold cycle (∆Ct) and calculating the relative gene expression according to the Protocol. Rationing and internal control were performed for the GAPDH household gene. Statistical control was performed relative to the control group. The selection of primers was carried out based on publicly available materials on the DNA and mRNA sequences of genes in the NCBI database using the Primer-BLAST program. For the CD4, forward primer was 5′-CCTCCTGCTTTTCATTGGGCTAG-3′ and reverse primer was 5′-TGAGGACACTGGCAGGTCTTCT-3′, for the CD8, forward primer was 5′-CCGTTGACCCGCTTTCTGT-3′ and reverse primer was 5′-CGGCGTCCATTTTCTTTGGAA-3′, for the CD19, forward primer was 5′-GGTACCGCCACCATGGCACCTCCTCGC CTCCTCTTC-3′ and reverse primer was 5′-AAGCTTG CCACCTGAGGATCACCTGGTGC-3′ and for the GAPDH, forward primer was 5′-GTGCCAGCCTCGTCCCGTAG-3′ and reverse primer was 5′-TTGCCGTGAGTGGAGTCATAC-3′. 

### 2.15. Statistical Analysis

Data are presented as mean ± standard deviation (SD) from at least six sets of measurements. Quantitative data were analyzed using ANOVA rank analysis of variance. *p* < 0.05 was accepted as being statistically significant.

## 3. Results and Discussion

### 3.1. Preparation and Analysis of Graphene Dispersion

The main difficulty in obtaining graphene-containing polymer materials is to achieve isotropy and to exclude the agglomeration of graphene in the entire volume of the polymer matrix [40]. Ultrasonic treatment and the use of amphiphilic stabilizers make it possible to simultaneously exfoliate graphene in an aqueous solution, as well as the assembly of hydrophobic graphene with a hydrophilic polymer matrix [41,42]. Therefore, in order to obtain aqueous graphene dispersions, we used Plu consisting of blocks of hydrophobic polypropylene oxide and hydrophilic polyethylene oxide groups, as well as PVP containing hydrophobic aliphatic and polar amide groups. It is noteworthy that PVP and Plu used in this work have been generally recognized as biocompatible, bioresorbable and widely applicable in medicine compounds [43,44].

**Figure 2:**
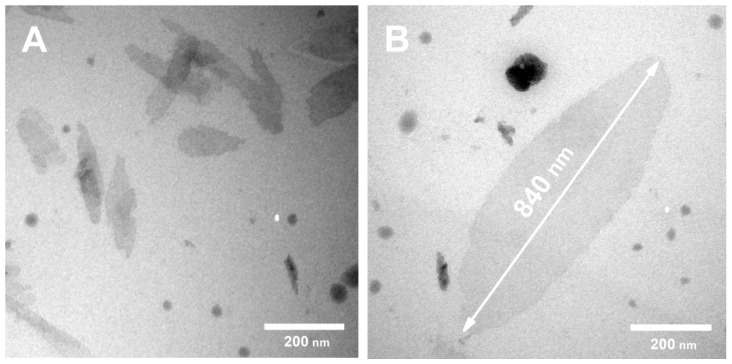
TEM-images of graphene particles, stabilized with PVP (**A**) and Plu (**B**). The particles of different sizes were obtained depending on the type of stabilizer and the duration of ultrasonic treatment. The duration of ultrasonic treatment of Plu-stabilized dispersions is 1–2 h, in case of PVP-stabilized it was 2–4 h.

The formation of dispersions with the highest graphene concentration was achieved at the concentration of the stabilizer being 10 mg/mL, independent of the stabilizer type. The following increase in the PVP/Plu concentration did not improve the outcome and was considered unnecessary. We also adjusted the duration of the ultrasonic treatment in order to provide the satisfactory interlayer cleavage, while avoiding the destruction of the graphene layers. When PVP was employed, we obtained 1.2 mg/mL dispersions of graphene with the lateral dimensions of 50–400 nm, according to TEM data (Figure 2), following a four-hour ultrasound treatment; while Plu provided 1.5 mg/mL dispersions of graphene with particle sizes of 150–1000 nm following two-hour ultrasound treatment. A further increase in the time of the ultrasonic treatment led to the destruction of the graphene particles, the size of which determined the electrical conductivity, as well as cell adhesion. All obtained dispersions were stable for 10–14 days and the number of layers ranged from one to four.

### 3.2. Formation and Analysis of Composite Films

Films were obtained by the solution casting method onto a horizontal substrate. To this purpose, CS was added to the graphene dispersions and acidified by lactic acid. Lactic and acetic acids were used as solubilizers; however, the most elastic and durable films were formed with lactic acid, presumably due to its higher acidity, which provides a more effective protonation of CS and its intermolecular interactions with the polymer matrix. On the basis of the obtained composite solutions, we produced elastic graphene-incorporated films using lactic acid as a solubilizer and PVP/Plu as a stabilizer.

**Figure 3:**
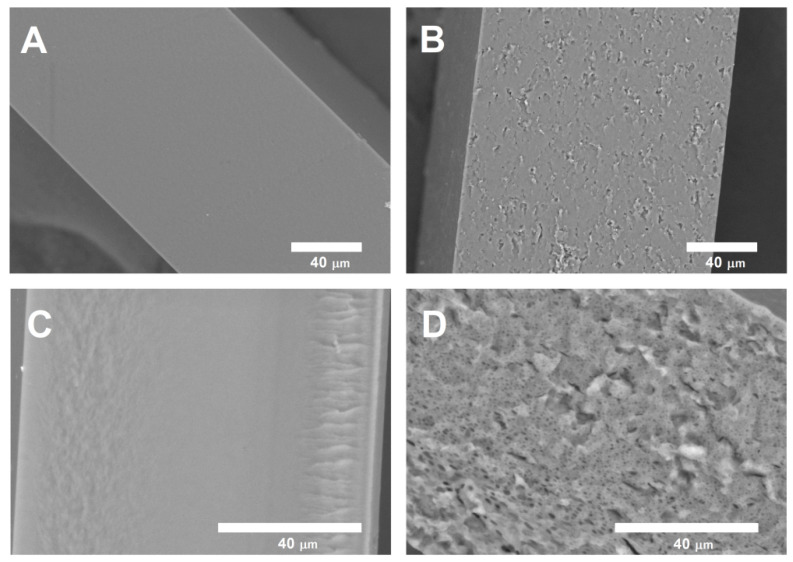
SEM-micrographs of the cross sections of films: (**A**) CS-PVP, (**B**) CS-PVP-G 1%, (**C**) CS-Plu, (**D**) CS-Plu-G 1%. Cross-sectional images show a rough internal structure of composite films associated with intermolecular interactions of the components.

**Figure 4:**
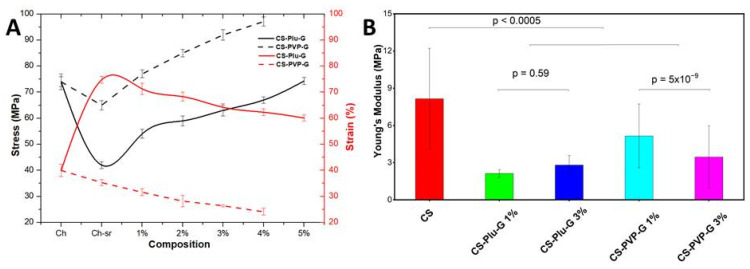
(**A**) Uniaxial tensile tests of CS, CS-PVP, CS-Plu and graphene-containing composite films. (**B**) Mechanical properties of CS/graphene composite films measured via nanoindentation.

According to SEM data (Figure 3), the inhomogeneities and agglomerates do not appear on the surface of the films, indicating the assembly of graphene with the polymer matrix. It can be seen from the cross-sectional images (Figure 4) that the introduction of graphene into the polymer matrix leads to a change in the morphology of the composites. Obviously, the cross sections of the composite films with graphene appear rougher than those of the films composed of pure chitosan. This is probably due to the formation of hydrogen bonds between CS and stabilizers, as well as the hydrophobic interactions of stabilizers and graphene. It could be noted that SEM images of composite films with other concentrations of graphene were almost the same.

Uniaxial tensile tests (Figure 4A) showed that the used stabilizers have a different effect on the dynamics of changes in the mechanical characteristics of the films. So, due to the low mechanical properties of the stabilizers, the strength is reduced, while the elongation at the break in the case of Plu increases by almost two times, which indicates a pronounced plasticizing effect of Plu, and the use of PVP, on the contrary, reduces the elongation at the break. For Plu-stabilized films, the addition of graphene lead to a further significant increase in strength, reaching a maximum value of 74.3 MPa for a CS-Plu-G of 5%. The addition of graphene to PVP-stabilized films also increased the mechanical strength, with the strongest CS-PVP-G 4% sample showing a limit value of 97.1 MPa. The elongation at the break of the films with an increase in the graphene content decreases for both stabilizers: from 74.75 to 60.1% for Plu-stabilized films and from 35.3 to 24.1% for PVP-stabilized films. This increase in strength and decrease in elongation at the break is explained by the intrinsic mechanical properties of graphene, which impart additional rigidity to the films.

According to nanoindentation data (Figure 4B), the Young’s modulus decreases due to the modification by stabilizers, mostly by Plu, which is explained by a change in the architectonics of the samples. A decrease in the Young’s modulus indicates a looser organization of the polymer network. An increase of the graphene amount in PVP-stabilized composite films is accompanied by an additional decrease in the Young’s modulus, which is explained by the increased roughness of the film surface.

**Figure 5:**
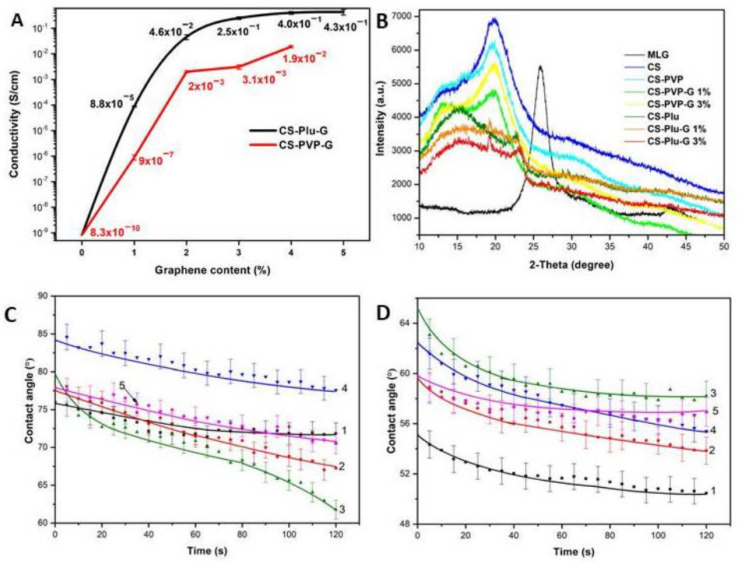
(**A**) Electrical conductivity of CS-PVP/Plu composite films containing exfoliated graphene. (**B**) XRD investigations did not reveal the patterns of MLG, indicating high degree of exfoliation of graphene. The static contact angle measurements of CS (1), CS-Plu-G 1% (2), CS-Plu-G 3% (3), CS-PVP-G 1% (4) and CS-PVP-G 3% (5) films in distilled water (**C**) and ethylene glycol (**D**).

It was expected that graphene, in addition to its mechanical characteristics, will affect the electrical conductivity of the materials. CS-PVP-G 1% demonstrates a conductivity of 9 × 10^−7^ S/cm, which is three orders of magnitude higher in comparison with pure CS [45]. In the case of CS-Plu-G 1%, the conductivity value of 8.8 × 10^−5^ S/cm is five orders of magnitude higher in comparison with pure CS. A further increase in the graphene content films leads to a gradual increase of electrical conductivity, which reaches a maximum value of 1.9 × 10^−2^ S/cm for the CS-PVP-G 4% and 4.3 × 10^−1^ S/cm for CS-Plu-G 5%. It should be noted that films with Plu demonstrated higher conductivity than those with PVP in each test performed at the same graphene content. This is explained by the larger lateral dimensions of Plu-stabilized graphene layers, as compared to graphene layers stabilized by PVP, which leads to an increase in electrical conductivity (Figure 5A).

At the 3% concentration of graphene, the electrical conductivity reached a plateau. In regard to this, in the consequent work, we performed an in-depth analysis of the samples with 1 and 3 % graphene contents.

Figure 5B represents the XRD analysis of pure CS film, MLG, CS-PVP, CS-Plu and composite films containing graphene. X-ray diffraction patterns of CS and CS-PVP films demonstrate a peak at 2θ = 21°, which is characteristic of chitosan. In addition, CS-PVP film and PVP-stabilized graphene composite films show peaks at 2θ = 13°, belonging to PVP [46]. From this, it can be concluded that the addition of PVP to CS does not lead to a significant change in the structure of the films. In case of CS-Plu film and graphene composites based on Plu, characteristics of Plu peaks at 2θ = 18° and 2θ = 24° are observed [47]. Moreover, the diffraction patterns show a displacement of the peak of CS from 21° to 16°. The displacement of the peak may occur due to the fact that Plu in the polymer mixture greatly reduces the degree of the crystallinity of CS due to its own high degree of disorder. It is noteworthy that according to the XRD data, the obtained composites do not demonstrate a peak at 26°, which is characteristic of MLG. It indicates that graphene has been exfoliated by ultrasonic treatment, as well as that there is no agglomeration of graphene layers during the fabrication of composite films.

The static contact angle of the film surface was measured to be <90° indicating a hydrophilic nature of all the modifications (Figure 5C). The measuring error was ~10%. A slight tendency to increase in the contact angle of water using PVP as a stabilizer of films can be observed. In case of Plu, the films show better hydrophilicity in comparison with a film of neat CS. The reason of this is in the nature of polymer stabilizers. In an aqueous solution PVP has a shape of a coil while Plu forms micelles. PVP binds to graphene via hydrophobic hydrocarbonic fragments. It leads to a decrease in the contact angle for the sample CS-PVP-G 3% due to the uniform interaction of PVP, CS and graphene [48]. For Plu, it is probably that the hydrophilic ethylene oxide sites of the stabilizer are located on the surface of the film based on CS-Plu-G 3%, which also leads to a significant decrease in the contact angle [49]. 

The measurements of the contact angle of ethylene glycol demonstrate better hydrophilicity (Figure 5D). There was a tendency of the contact angle to rise with an increase in the concentration of graphene in the films, which is associated with its hydrophobicity.

Table 1 shows the calculated values of the surface energy of the samples by the OWRK method based on the measured static contact angles. The values of surface energy are unique for each sample. In general, the use of stabilizers increases the total energy of the sample surface, which is favorable for cell adhesion. Cell adhesion and proliferation processes are known to depend on the chemical composition of the surface, roughness, free surface energy, charge and hydrophilicity [50,51,52,53]. In particular, Hallab et al. have found that the polar component of free surface energy is responsible for the cell adhesion strength [54].

### 3.3. Cytotoxicity In Vitro

**Figure 6:**
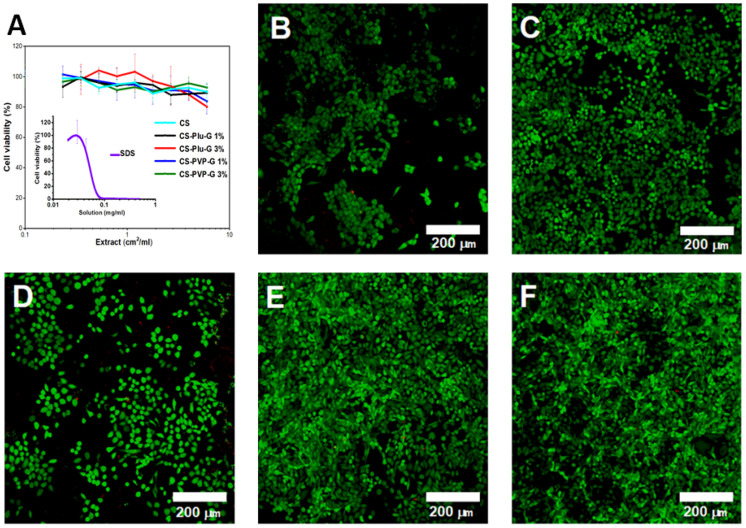
(**A**) Cytotoxicity of composite films established by MTT-test with SDS. The graphs show that films do not release cytotoxic components into cell culture medium in comparison with SDS. Confocal laser scanning microscopy of 3T3 murine fibroblasts stained with calcein-AM (green) and ethidium homodimer-1 (red) after 72 h of culturing on the sample surfaces and a glass coverslip (control): (**B**) CS, (**C**) CS-Plu-G 1%, (**D**) CS-Plu-G 3%, (**E**) CS-PVP-G 1%, (**F**) CS-PVP-G 3%. Life–dead method has shown that films are amiable for cell culturing. The scale bar represents 200 µm.

In order to evaluate the biocompatibility of the composite films, we performed in vitro and in vivo testing. According to the literature, scaffolds based on graphene can be either biocompatible or toxic to living cells. The response of cells to these nanomaterials depends on various factors, including their layer number, lateral size, dose, purity, surface chemistry and hydrophilicity [55]. In our study, the MTT-test showed that the extracts from the films did not compromise metabolic activity and proliferation of murine fibroblasts 3T3 (Figure 6A). According to the ISO 10993-5-2011: “Medical Devices. Biological Evaluation of Medical Devices, Part 5”, biomaterial is considered non-cytotoxic if cell viability is ≥70% of the blank. SDS used as a positive control caused remarkable cell death (IC50~0.05 mg/mL).

In order to determine the confluence, viability and morphology of mouse fibroblasts 3T3-cultured on the film surface, we performed the staining of the cells with Calcein-AM (green, staining live cells) and ethidium homodimer (red, staining dead cells). A high number of living cells were observed on the films after 72 h in culture, with very few red inclusions, as revealed in Figure 6B–F. The confocal microscopy showed that all polymeric matrices provided satisfactory cell adhesion and proliferation, with the total cell death not exceeding 1%. However, there was a moderate tendency for cells to be rounded (Figure 6B–F). The use of stabilizers had favorable effects, most pronounced with PVP. The cells took a physiologic spindle-like configuration and also provided a uniform distribution with a typical fibroblast organization of concentric patterns. In earlier studies, biomaterials based on CS [56,57] and graphene [58,59] were shown not to exhibit cytotoxic properties. In CS-Plu-G 3%, cell adhesion was lower compared to other samples despite the high total surface energy. This can be explained by the complex interaction of the biomaterial with the cells. Plu forms a protective layer on the cell membrane, which has a significantly lower hydrophobicity and reduce surface attachment [60].

### 3.4. Subcutaneous Implantation in Wistar Rats

Microscopic imaging of histological sections of encapsulated implants showed a similar pattern in all groups: under the layer of subcutaneous fat was an implant surrounded by a connective tissue capsule.

**Figure 7:**
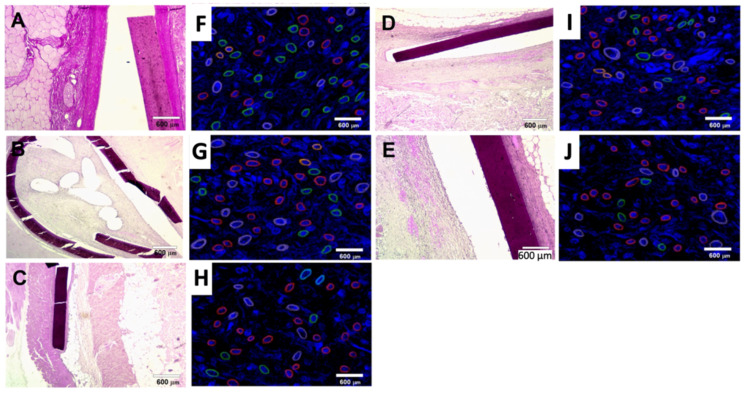
Histological study and results of an immunofluorescent study of the films following 30 days of subcutaneous implantation in rats under hematoxylin-eosin staining: (**A**) CS; (**B**) CS-Plu-G 1%; (**C**) CS-Plu-G 3%; (**D**) CS-PVP-G 1%; (**E**) CS-PVP-G 3%. Chemical modification of the films by graphene and stabilizers reduces the risk of implant destruction and inhibit leukocyte infiltration. Results of an immunofluorescent study of CD4+ marker expression for CD4+ T-lymphocytes (red), CD8+ for CD8+ T-lymphocytes (green), CD68 for macrophages (pink), CD20 for B lymphocytes (orange) and CD138 for plasma cells (blue) in CS (**F**), CS-Plu-G 1% (**G**), CS-Plu-G 3% (**H**), CS-PVP-G 1% (**I**) and CS-PVP-G 3% (**J**) groups. Chemical modification of the films by graphene and stabilizers has led to decrease in the pro-inflammatory lymphocytes and an increase in the anti-inflammatory lymphocytes. The scale bar represents 600 µm.

It was noted that the extent of the destruction of the film matrices varied depending on the chemical composition of the material (Figure 7A–E). The use of graphene as a filler reduced film deformation during animal life. There is evidence in the literature that such materials, with a high degree of deacetylation of CS (>90%), become brittle and degrade due to the appearance of a large number of amino groups in the polymer chain [61]. Hydrogen bonding to the graphene surface is facilitated and, therefore, the stiffness of the material is increased. Analogs with a lower degree of deacetylation, on the contrary, are characterized by the ability to exhibit forced elastic deformation, which provides a spatial orientation of the material during implantation, which helps to maintain the integrity of the implant and, accordingly, prevent injury to surrounding tissues.

The thickness of the woven capsule also varied throughout. In the structure of connective tissue, vessels such as arterioles, capillaries and venules were traced. The morphological organization of the capsule was also heterogeneous: a compact connective tissue containing a large amount of collagen adjoined the implant directly; around the periphery of the capsule was a layer of looser connective tissue having collagen, elastic fibers.

The cellular composition of the surrounding tissues was characterized by the predominance of cell elements mainly of the fibroblastic series. Giant cells of foreign bodies, called macrophages, which are generally characteristic of the natural biodegradation of materials implanted into the recipient’s organism, are revealed closer to the implant from the inner surface [62]. In addition, implantation of most CS films caused moderate leukocyte infiltration, expressed at the sites of the greatest material segmentation (Figure 7F–J). The development of the inflammatory response in this case could be both the result of the lifetime traumatization of the overimplantation of soft tissues due to the fragility of the implants and the direct stimulation of leukocyte migration to the postoperative wound area. In contrast, the saturation of CS films with PVP and Plu contributed to some reduction in leukocyte migration in the implantation area, but areas of leukocyte accumulation were also observed in these micropreparations. The above suggests that the composition of the films affects their biocompatibility.

### 3.5. Immunofluorescence Study of Reactive Infiltrates

**Figure 8:**
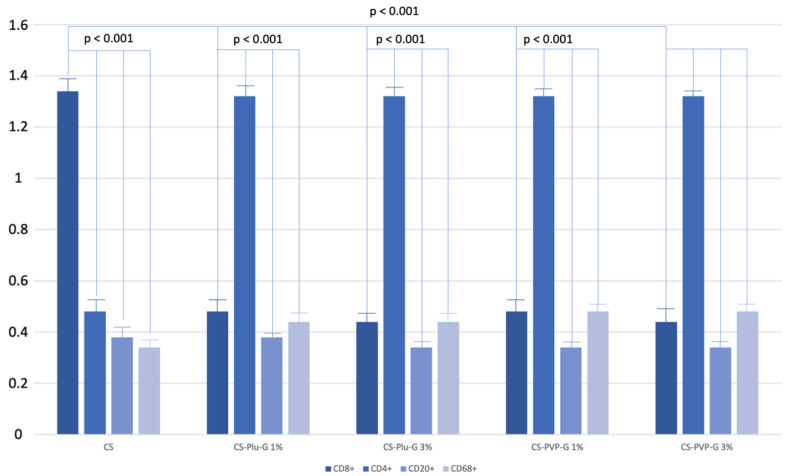
Polymerase chain reaction results to detect expression activity of CD8+, CD4+, CD20+ and CD68+ markers in CS, CS-Plu-G 1%, CS-Plu-G 3%, CS-PVP-G 1% and CS-PVP-G 3% sample groups.

In most cases, as discussed above, moderate leukocyte infiltration of a predominantly focal nature was observed. It is important to note that the largest number of leukocyte clusters was observed in the area of segmentation of the implanted material. An immunofluorescence study with markers for CD4+ T-lymphocytes, CD8+ T lymphocytes, macrophages, B-lymphocytes and plasma cells, together with PCR were used to type the leukocyte populations and the general assessment of the nature of the immune response. It was found that there are significantly more cells found in leukocyte infiltrates in CS than infiltrates in the CS-Plu-G 1% (*p* = 0.001), CS-Plu-G 3% (*p* = 0.003), CS-PVP-G 1% (*p* = 0.024) and CS-PVP-G 3% (*p* = 0.002) groups (Figure 8). When considering the intra-group heterogeneity of leukocyte infiltrates, it was found that CD8+ T-lymphocytes significantly predominate in the CS group, making up about 34.28 ± 2.48% of the cell population (two-way ANOVA, *p* < 0.001), while in groups with samples saturated with PVP and PLU, CD4+ T-lymphocytes with 32.26 ± 2.38% and 32.38 ± 4.18%, respectively, predominated in the cell populations (two-way ANOVA, *p* < 0.001 for both). The content of the B-lymphocytes and plasma cells was extremely low in all groups, while macrophages were in second place in the frequency of occurrence in the CS-Plu-G 1% (*p* < 0.001), CS-Plu-G 3% (*p* < 0.001), CS-PVP-G 1% (*p* < 0.001) and CS-PVP-G 3% groups (*p* < 0.001) and third in the CS group (*p* < 0.001, Figure 8).

Therefore, we concluded that the chemical modification of implants has led to a decrease in the severity of the proinflammatory potential of reactive leukocyte infiltrates at the site of implantation. There was a decrease in the quantitative content of the pro-inflammatory compartment of the lymphocytic T cell population and an increase in the anti-inflammatory link. In general, the results obtained show a favorable effect of the modifications carried out on the engraftability properties of the embedded materials.

## 4. Discussion

Modern tissue engineering techniques focus on the regeneration and repair of tissue by creating scaffolds on which stem cells proliferate and form new tissues. Biocompatible and biodegradable polymers can be used to obtain scaffolds. 

In this article we prepared electrically conductive nanocomposite graphene/chitosan films for conductive tissue engineering. To obtain composite films, graphene dispersions are usually preliminarily prepared with the subsequent addition of a polymer. However, it is very difficult to obtain dispersions from pristine graphene. To solve this problem Natalia Wrońska et al. [63] prepared the graphene dispersions using a modified form of graphene—GO. Unfortunately, GO is a dielectric, which significantly limits the use of composites based on it and does not allow the electrical stimulation of stem cells, which is very important for the growth of cardiac and nerve tissues. Another paper [64] suggests the use of rGO, which has electrical conductivity, but in order to obtain it, toxic solvents are usually used, which can negatively affect the stem cells. In our work, we prepared the graphene dispersion using the biocompatible surfactants PVP and Plu, stabilizing the graphene particles to prevent the agglomeration of a filler. The obtained films based on preparing dispersions demonstrated different results of the tensile strength and elongation at the break. In our work, we obtained the values of the tensile strength of the films up to 85 MPa. Mounir El Achaby et al. [65] prepared the composites, consisting of GO instead of pristine graphene. The obtained films showed values of tensile strength reaching 125.5 MPa for CS-PVP-G 2% film. In both works, an increase in tensile strength can be observed with the addition of a graphene filler. However, the addition of GO leads to a more pronounced increase in strength, which is explained by interactions of -OH groups of GO and -NH_2_ groups of CS. It is noteworthy that composites with pristine graphene demonstrated more pronounced elongation at the break in comparison with composites with GO. The use of lactic acid instead of acetic acid led to the formation of more flexible films, which affected the results of elongation. However, GO-modified composites were not electrically conductive. A. Barra et al. [66] obtained electrically conductive CS-rGO composite films, which reach a maximum value of 7 × 10^−3^ S/cm for CS-rGO50. In comparison with this work, we were able to achieve electrical conductivity values up to 4.3 × 10^−1^ S/cm. Thus, the use of biocompatible surfactants such as PVP and Plu makes it possible to obtain graphene/chitosan composites with high values of electrical conductivity.

According to the literature, the chemical composition of the scaffold, as well as the biomimetic simulation of the microenvironmental characteristics, can contribute to cell growth, vascularization, nutrient delivery and the transport of metabolites [67]. Our study shows that hydrophilicity and biocompatible scaffold properties did not significantly deteriorate with the chemical modification of chitosan films. The film structure remained homogeneous, thus providing favorable conditions for uniform adhesion and cell proliferation. The inclusion of graphene and stabilizers in the polymer composition helps to overcome the main structural and mechanical constraints of the scaffold. The obvious advantage of developed films is the technical simplicity of creating the desired shape and ease of surgical manipulations. Such films with an improved structure and performances can be a good platform for replacing the heterogeneity areas formed by fibrous tissue in postinfarction cardiosclerosis. Previously [68], it was described that the most structurally and functionally optimal three-dimensional constructs for cardiac reconstruction have been created using electrical stimulation, probably because the natural environment provides the best conditions for cell adhesion, alignment and coupling. New investigations in this field are aimed at developing conductive hydrogels for intracardiac applications. However, the mismatch of stiffness and conductivity discrepancies between hydrogels and cardiac tissue do not maintain the stable structure of the implantable material under continuous contractional forces of the beating heart [69]. Our results have shown that proper electrical conductivity and stable mechanical properties contributed to cellular growth and supported functional cell activity. The electrophysiological properties of scaffolds are extremely important for conducting an electrical signal between cells and can be used in the future for removing blocks of different localizations (atrioventricular, intraventricular block, etc.).

## 5. Conclusions

To sum up, a method to obtain graphene dispersions for the formation of biomaterials based on chitosan was developed. The introduction of graphene into the polymer matrix of chitosan led to an improvement of mechanical strength and electrical conductivity. The resulting film materials were hydrophilic and did not emit cytotoxic components. 

It was shown that selected ultrasonic treatment conditions (2 h for Plu, 4 h for PVP, 100 W, 35 kHz) made it possible to obtain highly concentrated (up to 1.5 mg/mL) dispersions which were stable for up to 2 weeks. It has been determined that the type of stabilizer had an effect on the duration of the ultrasonic treatment of dispersions, which led to a decrease in the sizes of the graphene particles. At the next stage of the research, we developed elastic films with a graphene content of 1–4 wt.% for films with PVP and 1–5 wt.% for films with Plu on the basis of the graphene dispersions and lactic acid solutions of chitosan. According to SEM and XRD, graphene was distributed in the films homogeneously and agglomerations of graphene particles were not observed. The composite films were hydrophilic and the contact wetting angle was less than 90°. We established that the mechanical properties, electrical conductivity of composite films and cell adhesion to their surface are determined not only by the content of graphene, but also by the nature of the stabilizer used. Thus, it was found that the use of Plu as a stabilizer led to the increase in the relative elongation of the samples by 2–2.5 times in comparison with the samples where the stabilizer is PVP. In addition, the electrical conductivity of the samples with Plu was an order of magnitude higher than with PVP at the same content of graphene, which is probably due to the size of the stabilized graphene layers.

All composite films provided the adhesion and proliferation of fibroblasts and the number of dead cells did not exceed 1%. It should be noted that the use of PVP as a stabilizer decreased the rounding of cells observed for Plu which may be related to the energy characteristics of its surface. The tissue response to implantation was characterized by the formation of a foreign-body-type reaction around the specimens with the development of granulation tissue and fibrosis. Capsule thickness depended on the specimen’s composition and did not exceed 1.5 mm. The chemical modification of the implants by stabilizers has led to a decrease in the severity of the proinflammatory potential of the reactive leukocyte infiltrates at the site of implantation. There was a decrease in the quantitative content of the pro-inflammatory compartment of the lymphocytic T-cell population and an increase in the anti-inflammatory link. This makes it possible to consider chitosan and graphene-based nanocomposite films as promising reconstructive materials for conductive tissue engineering. Our future research will focus on the use of electrically conductive scaffolds in vivo, in models of myocardial infarction.

## Figures and Tables

**Table 1:** Surface energy of CS/graphene composite films.

Sample	Wetting Edge Angle, θ, Degrees (Water/Ethylene Glycol)	Surface Energy and Its Components, mJ/m^2^		
		Polar, ơ^p^_s_	Dispersive, ơ^d^_s_	Total Energy, ơ^p^_s_ + ơ^d^_s_= ơ_s_
CS	(73 ± 1.5)/(52 ± 1)	15 ± 2	17 ± 1	32 ± 1
CS-Plu-G 1%	(72 ± 3.5)/(56 ± 1.5)	19.5 ± 2.5	11.5 ± 2	31 ± 2
CS-Plu-G 3%	(70 ± 4.5)/(59 ± 2)	26 ± 4	7 ± 2	33 ± 3
CS-PVP-G 1%	(81 ± 2.5)/(58 ± 2)	7 ± 3	20 ± 2	28.5 ± 2.5
CS-PVP-G 3%	(74 ± 2)/(57 ± 1)	17 ± 3	13 ± 2	30 ± 2

## Data Availability

The data presented in this study are available on request from the corresponding author.

## Supplementary Materials

The following supporting information can be downloaded at: https://www.mdpi.com/article/10.3390/polym14183792/s1, Table S1: Composition of graphene films; Figure S1: No-primary-control reactions image for the immunostaining procedures in the study.
