# Supplementary material for: Fabrication of Conductive Tissue Engineering Nanocomposite Films Based on Chitosan and Surfactant-Stabilized Graphene Dispersions
Source: Polymers (Basel). 2022 Sep 10;14(18):3792. doi: 10.3390/polym14183792 (PMC9503515; doi:10.3390/polym14183792)
Supplement: Supplementary file 1 [file polymers-14-03792-s001.zip › polymers-1875061-supplementary.pdf]

polymers

MDPI

Supporting information

Fabrication of Conductive Tissue Engineering Nanocomposite Films Based on Chitosan and Surfactant-Stabilized Graphene Dispersions

Aleksandr S. Buinov [1], Elvira R. Gafarova [2,3,*], Ekaterina A. Grebenik [2], Kseniia N. Bardakova [2,4], Bato Ch. Kholkhoev [1], Nadezhda N. Veryasova [2], Pavel V. Nikitin [2,3], Nastasia V. Kosheleva [2,5], Boris S. Shavkuta [2], Anastasia S. Kuryanova [6], Vitalii F. Burdukovskii [1] and Peter S. Timashev [2,3,6,7]

1 Baikal Institute of Nature Management, Siberian Branch, Russian Academy of Sciences, Marii Sakh'yanovoi st. 6, 670047 Ulan-Ude, Russia
2 Institute for Regenerative Medicine, Sechenov University, Trubetskaya st. 8-2, 119991 Moscow, Russia
3 World-Class Research Center "Digital Biodesign and Personalized Healthcare", Sechenov First Moscow State Medical University, Trubetskaya st. 8-2, 119991 Moscow, Russia
4 Institute of Photonic Technologies, Federal Scientific Research Centre "Crystallography and Photonics", Russian Academy of Sciences, Pionerskaya st. 2, 142190 Troitsk, Russia
5 FSBSI "Institute of General Pathology and Pathophysiology", Baltiyskaya st. 8, 125315 Moscow, Russia
6 Semenov Federal Research Center of Chemical Physics, Russian Academy of Sciences, Kosygina st. 4, 119991 Moscow, Russia
7 Chemistry Department, Lomonosov Moscow State University, Leninskiye Gory 1-3, 119991 Moscow, Russia
* Correspondence: gafarova_e_r@staff.sechenov.ru

Table S1. Composition of graphene films.

Sample	wt %		
	CS	St	graphene
CS-St-G 1%	**65.67**		**1**
CS-St-G 2%	**64.67**		**2**
CS-St-G 3%	**63.67**	**33.33**	**3**
CS-St-G 4%	**62.67**		**4**
CS-St*-G 5%	**61.67**		**5**

St – stabilizer (Plu or PVP).
St* - only Plu.
Total weight of film was 600 mg.

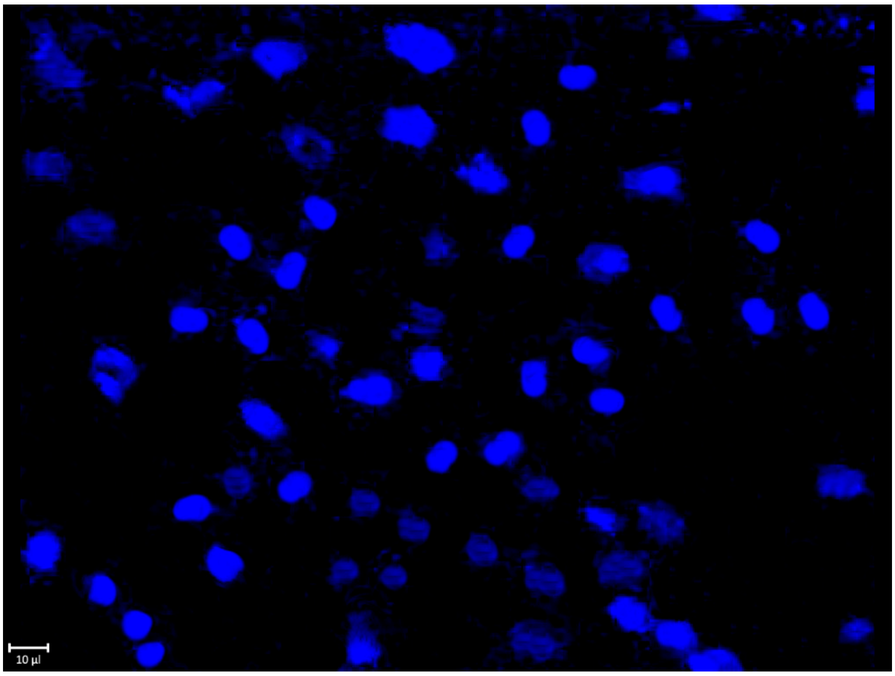

Figure S1. No-primary-control reactions image for the immunostaining procedures in the study.
